# University of Maryland Medical School

**Published:** 1881-03

**Authors:** 


THE UNIVERSITY OF MARYLAND MEDICAL SCHOOL held its Seventy-fourth Annual Commencement on Thursday the 3rd instant, and granted its Diploma to seventy-three of its students. The degree of M. D., was conferred upon the graduates by Hon. S. T. Wallis, LL. D., Provost of the University. Prof. I. Edwin Michael, M. D., delivered an earnest and instructive address to the graduates.

